# Supplementary material for: Inoculation with Rhizophagus irregularis Does Not Alter Arbuscular Mycorrhizal Fungal Community Structure within the Roots of Corn, Wheat, and Soybean Crops
Source: Microorganisms. 2020 Jan 7;8(1):83. doi: 10.3390/microorganisms8010083 (PMC7023141; doi:10.3390/microorganisms8010083)
Supplement: Supplementary file 1 [file microorganisms-08-00083-s001.zip › supplementary.pdf]

Supplementary materials

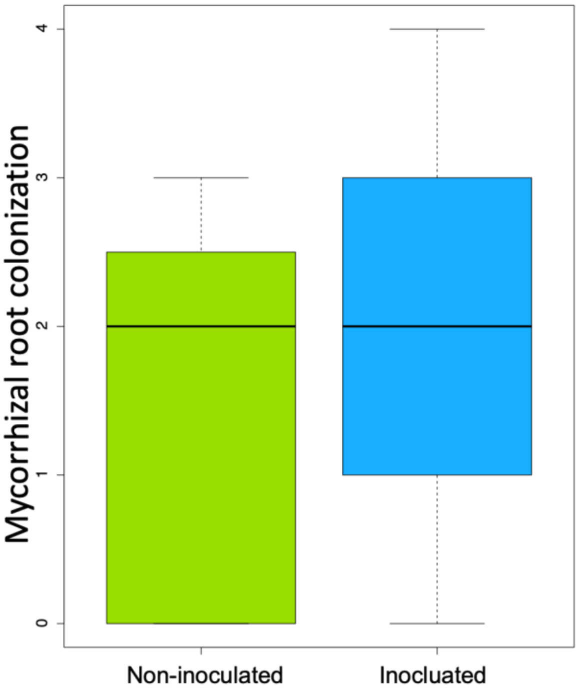

Figure S1. on inoculated and non-inoculated samples. AMF inoculation didn't have any significant difference (*p*-value>0.05).

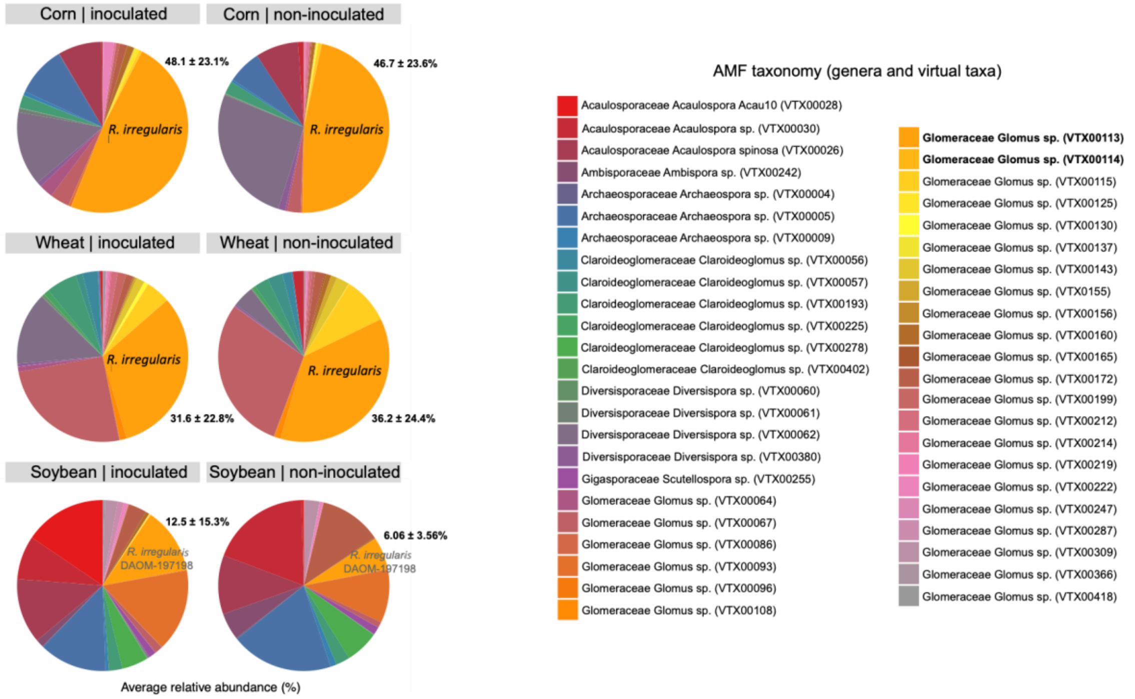

Figure S2. Abundance of AMF taxa in inoculated and non-inoculated plots of corn, wheat and soybean. *R. irregularis* represented by VTX00113 and VTX0011, dominated AMF diversity in corn and wheat.

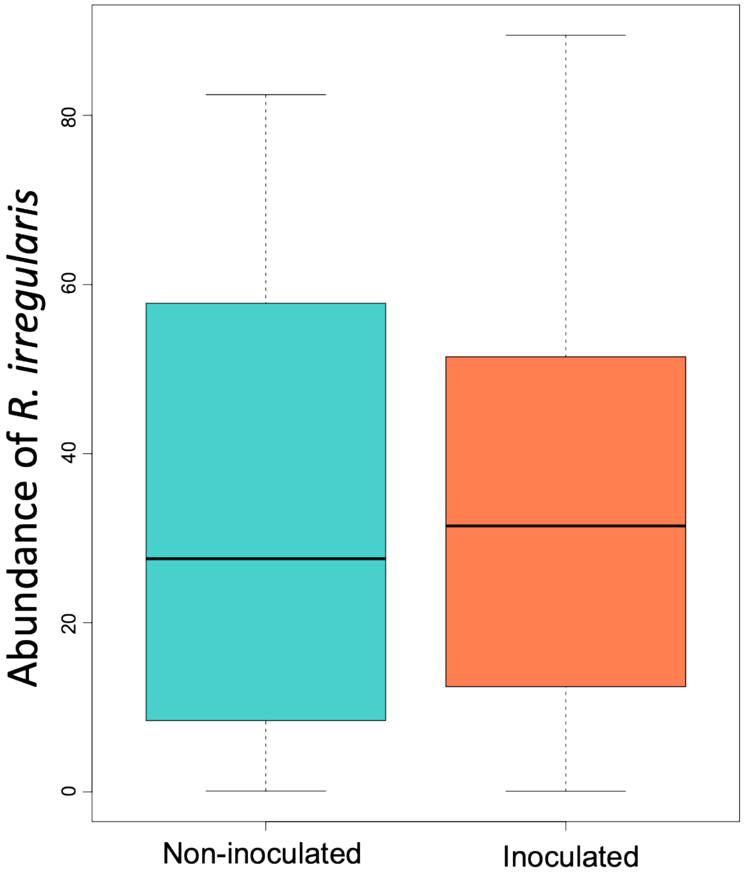

Figure S3. No significant differences in the relative abundance of *R. irregularis* in the roots of inoculated and non-inoculated soils (*p=0.173*).

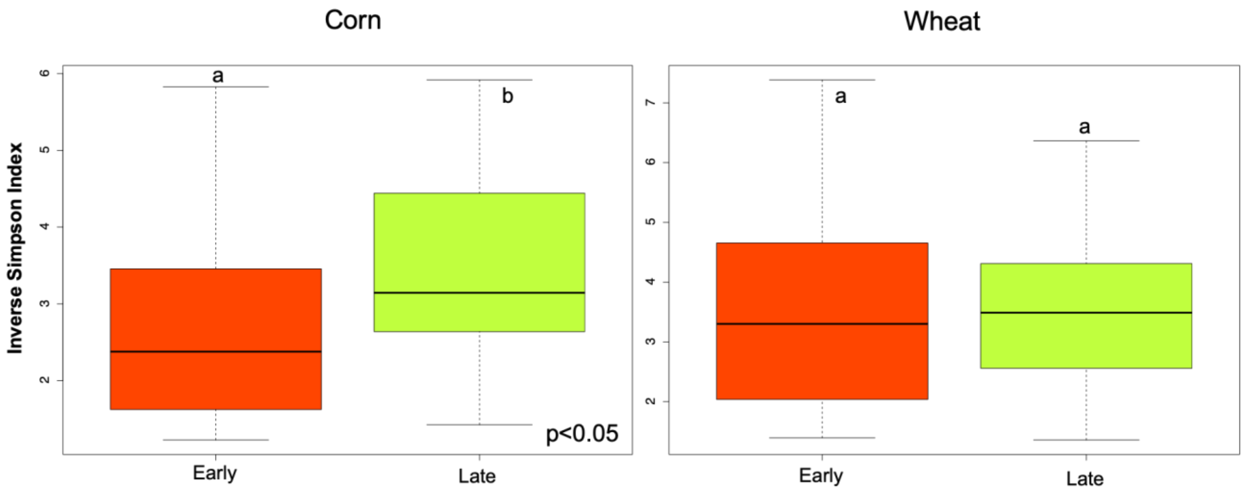

Figure S4. Corn roots at a late growth-stage had a higher alpha-diversity than corn roots at an early growth-stage (*p=0.037*), while no significant difference was observed between growing stages in wheat.

Table S1. Relative abundance can taxonomy of all 414 OTUs of AMF.
Link at GiHub: https://github.com/seb951/Premier_Tech_inoculation/blob/master/results/TableS1_OTU_VT_abundance.txt
